# Cardiac parasympathetic index identifies subjects with adult obstructive sleep apnea: A simultaneous polysomnographic-heart rate variability study

**DOI:** 10.1371/journal.pone.0193879

**Published:** 2018-03-08

**Authors:** Maria Salsone, Basilio Vescio, Andrea Quattrone, Ferdinando Roccia, Miriam Sturniolo, Francesco Bono, Umberto Aguglia, Antonio Gambardella, Aldo Quattrone

**Affiliations:** 1 Institute of Bioimaging and Molecular Physiology, National Research Council, Germaneto, Catanzaro, Italy; 2 Biotecnomed S.C. aR.L., Catanzaro, Italy; 3 Institute of Neurology, Department of Medical Sciences, University Magna Graecia, Catanzaro, Italy; 4 Institute of Rehabilitative Cardiology, Department of Medical Sciences, University Magna Graecia, Catanzaro, Italy; 5 Neuroscience Center, University Magna Graecia, Germaneto, Catanzaro, Italy; University of Rome Tor Vergata, ITALY

## Abstract

**Objective:**

To evaluate circadian fluctuations and night/day ratio of Heart Rate Variability (HRV) spectral components in patients with obstructive sleep apnea (OSA) in comparison with controls.

**Participants and methods:**

This is a simultaneous HRV-polysomnographic (PSG) study including 29 patients with OSA and 18 age-sex-matched controls. Four patients with OSA dropped out. All participants underwent PSG and HRV analysis. We measured the 24-hour fluctuations and the night/day ratio of low frequency (LF) and high frequency (HF) spectral components of HRV in all subjects and controls. The LF night/day ratio was termed the cardiac sympathetic index while the HF night/day ratio was termed the cardiac parasympathetic index.

**Results:**

All twenty-five OSA patients were PSG positive (presence of OSA) while 18 controls were PSG negative (absence of OSA). There was no significant difference in LF and HF 24-hour fluctuation values between OSA patients and controls. In OSA patients, LF and HF values were significantly higher during night-time than day time recordings (p<0.001). HF night/day ratio (cardiac parasympathetic index) accurately (100%) differentiated OSA patients from controls without an overlap of individual values. The LF night/day ratio (cardiac sympathetic index) had sensitivity of 84%, specificity of 72.2% and accuracy of 79.1% in distinguishing between groups.

**Conclusions:**

The cardiac parasympathetic index accurately differentiated patients with OSA from controls, on an individual basis.

## Introduction

Obstructive sleep apnea (OSA) is a common sleep-related breathing disorder affecting at least 2–4% of the adult population [1,2]. It is characterized by repetitive collapse of the upper airway during sleep resulting in increased arousals, sleep fragmentation and decreased oxygenation [3]. Clinically, OSA is defined by the occurrence of symptoms leading to excessive daytime sleepiness (EDS), fatigue and mood problems [4,5]. OSA is also associated with impairment of cognitive function [6] such as decreased concentration, memory and executive dysfunctions thus exercising a negative effect on quality of life. Polysomnography (PSG) is currently the standard diagnostic test for the diagnosis of OSA [7], although it has several limitations as the high expense and inconvenience of an overnight sleep study. The use of PSG or home sleep apnea testing with a technically adequate device, is strongly recommended by the Task Force for the diagnosis of OSA in the uncomplicated adult patients with signs and symptoms suggestive of OSA [7] thus supporting the emerging role of portable monitoring in the OSA diagnosis.

The autonomic nervous system (ANS) plays an important role during sleep. Indeed, patients with autonomic dysfunction may have sleep disorders whereas patients with untreated sleep disorders may present features suggestive of autonomic dysfunction [8]. The autonomic impact of OSA has been discussed by Somers et al. [9], who analyzed the link between sleep apnea and cardiovascular disease and the effect of sleep apnea treatment on cardiovascular disease and clinical endpoints [9]. Others authors [10,11], have also found that in OSA patients repeated episodes of apnea or hypopnea can impact on ANS leading to cardiovascular consequences.

A simple and non invasive tool for investigating the cardiac autonomic system is heart rate variability (HRV) analysis [12]. There is well-documented evidence of short-term HRV analysis. In OSA patients, some authors [13] have demonstrated that LF (low frequency), VLF (very low frequency) and HF (high frequency) were strongly correlated with PSG diagnostic indices, whereas others found a significant relationship between HRV parameters and severity of OSA [14]. Some of these studies, however, were retrospective and thus did not analyze HRV and PSG parameters simultaneously.

Some authors [15] have explored HRV parameters during sleep stages in OSA subjects indicating that sympathovagal modulation may be impaired in these patients with parasympathetic activity tending to increase over the night.

Finally, only two studies [16,17] have been carried out in long term conditions (24-hour period). Some authors [16] found that the circadian rhythm of LF and HF spectral components of HRV analysis significantly differentiated patients with severe OSA from those with mild OSA and controls, whereas others [17] reported that LF values of OSA patients were significantly higher than the controls.

No report, however, has demonstrated the usefulness of HRV parameters in differentiating subjects with OSA from those without OSA on an individual basis. A recent study [18], reported that night/day ratios of the sympathetic and parasympathetic activities accurately differentiated patients with REM sleep behaviour disorder (RBD) from those without RBD on an individual basis thus indicating that these ratios may be potential tools for screening REM sleep disorders.

In the current study, using a combined simultaneous HRV-PSG recording, we investigated the circadian autonomic fluctuations and the night to day ratios of HRV spectral components in patients with OSA compared to controls. We also evaluated the accuracy of LF and HF night/day ratios in differentiating subjects with OSA from those without OSA.

## Materials and methods

### Participants

A total of 29 patients with OSA and 18 age-sex-matched controls were consecutively enrolled in the current study from the Neurology Unit of the University “Magna Graecia” of Catanzaro from January 2016 to March 2017. Before inclusion in the study, written informed consent was obtained from all participants. The study was approved by the Ethical Committee of the University ‘Magna Graecia’ of Catanzaro according to the Helsinki Declaration.

Patients with OSA were defined according to clinical guidelines for OSA diagnosis [7] which was based on a comprehensive sleep evaluation [7] including features suggestive of OSA such as habitual loud snoring, witnessed apnea, gasping or choking at night and excessive daytime sleepiness (EDS) not explained by other factors. Assessment of sleepiness severity was evaluated in all participants using the Epworth Sleepiness Scale (ESS) [19] which was completed by unassisted subjects following brief instructions provided by the physician. An ESS score ≥ 11 was considered suggestive of EDS [20]. In all participants we also evaluated medical conditions associated with the increased risk for OSA such as body mass index (BMI) ≥30 kg/m^2^ and hypertension. Four of the 29 patients with OSA dropped out. For the remaining 25 a complete neurological examination was performed to rule out the presence of other neurological disorders. In all patients, the presence of OSA on polysomnography (PSG) was required in order to confirm the clinical diagnosis [7]. Patients with PSG negative for OSA were classified as controls.

Exclusion criteria for all participants consisted of the presence of sleep disorders such as periodic leg movements during sleep (PLMS) and RBD detected by polysomnography and other comorbidities known to affect the autonomic nervous system and to interfere with autonomic evaluations such as cardiac arrhythmias, diabetes mellitus, pulmonary disorders and thyroid diseases.

### Polysomnography

All participants underwent eight-hour nocturnal monitoring (from 10 PM to 6 AM) using fully attended polysomnography (PSG) in a laboratory setting (type 1) [21]. We instructed each participant not to ingest alcohol or caffeine and not to do prolonged exercise on the day of the monitoring. The PSG was performed using Xltek^®^ EEG Systems. During the PSG, we collected physiologic signals required for evaluating OSA [1] by recording biopotentials (electroencephalogram, electromyogram, electrooculogram), airflow (thermal sensors), qualitative recordings of respiratory effort (piezo-sensors) and oxygen saturation (pulse oxymetry). Apnea was defined as the absence of respiration for more than 10 s. Hypopnea was defined as reduction of over 50% of the oro-nasal flow amplitude for at least 10 s resulting in a decrease in oxygen saturation of > 4% and/or arousal. Hypopnea was defined as being obstructive if there was upper airway resistance such as snoring, paradoxical motion in the respiratory bands, or inspiratory flow limitation on nasal pressure signal [1]. The apnea-hypopnea index (AHI) was calculated as the number of apneas and hypopneas events recorded during the study per hour of the sleep. OSA severity was defined as mild for AHI ≥ 5 and <15, moderate for AHI ≥15 and ≤ 30, and severe for AHI > 30/hr [20]. According to AHI, we stratified our patients with OSA patients as mild, moderate and severe. Sleep stages were scored according to American Academy of Sleep Medicine Manual for the scoring of sleep and associated events [22].

### Heart rate variability analysis

Autonomic control of heart rate (HR) was obtained in all participants by heart rate variability (HRV) analysis [23]. R-R intervals were taken from the 24-hour ECG recording (ECG/HRV device: Mega Electronics Emotion Faros 180°, 2-leads wearable ECG- HRV monitor). The sampling rate for ECG was set at 500Hz. The electrodes were placed approximately along the electrical axis of the heart. The optimal placement of the electrodes followed the locations of electrodes RA and V5 in the Mason-Likar modification of the standard 12 lead ECG: the negative electrode was placed on the right infraclavicular fossa (just below the right clavicle), and the positive electrode on the left side of the chest, below the pectoral muscle on the left anterior axillary line [18].

The RR signal (tachogram) was processed using PhysioNet HRV Toolkit base functions, integrated in Matlab^®^. Time series acquisition was then divided into 10-minute epochs, with an overlapping of 5 minutes, for frequency analysis. Each epoch was processed with means of the Lomb-Scargle algorithm, which computes the power spectrum of unevenly sampled data. LF (low frequency, sympathetic system) power was taken in the 0.04–0.15 Hz frequency band, while HF (high frequency, parasympathetic system) power was taken in the 0.15–0.4 Hz band as previously described in detail [18]. Detrended fluctuation analysis, information-based similarity, estimate of largest Lyapunov exponent, multiscale entropy analysis and multifractal analysis were performed by means of the PhysioNet open source libraries for the nonlinear analysis of time series [18].

### Study design

Participants underwent a circadian (24-hour) HRV recording (from 9 AM to 9 AM of the next day). During the night-time (from 10 PM to 6 AM) participants underwent a combined simultaneous HRV-PSG recording. During the diurnal registration (from 9 AM to 10 PM and from 6 AM to 9 AM of the next day), participants were independent in their activities [18]. The mean values of the spectral components of HRV (LF and HF) were calculated for the circadian 24-hour period, and during the night time and daytime periods. For all participants, we also calculated night-to-day ratios for both LF (cardiac sympathetic index) and HF (cardiac parasympathetic index) spectral components as previously described in detail [18]. All subjects stopped taking any anticholinergic, antidepressant, sympathomimetic, or parasympathomimetic medications *72* hours before HRV testing.

### Statistical analysis

Differences in sex, sleep evaluation, hypertension and abnormal ESS score distribution were assessed using of the Fisher’s exact test. The Shapiro-Wilk test was used to check for normality before performing comparisons. The two sample t-test and the Mann-Whitney U test were used to assess differences between OSA patients and controls for continuous and scale variables. The one-sample t-test and the Wilcoxon signed rank test were used to compare cardiac indexes, together with night and day values of LF and HF power within OSA patients and controls. Sensitivity, specificity and accuracy were determined in order to differentiate OSA patients and controls by evaluating optimal cutoffs for the Cardiac Parasympathetic Index and the Cardiac Sympathetic Index on the Receiver Operating Characteristic (ROC) curves. The optimal cut-off levels were defined as the values with the highest sum of sensitivity and specificity. Differentiating properties of the Cardiac Parasympathetic and Cardiac Sympathetic Indexes were compared using the McNemar’s test. Spearman’s rank correlation was used to test the association between ODI, AHI and P-index (Cardiac Parasympathetic Index) and a linear relationship was found between ODI, AHI and the logarithm of P-index by means of Pearson’s correlation test. Statistical analysis was performed with R Statistical Software (R for Unix/Linux, version 3.1.1, the R Software Foundation for Statistical Computing, 2014).

## Results

Four of the 29 patients with OSA included in the study dropped out. Demographic, clinical and electrophysiological data of patients with OSA and controls are shown in Table 1. The total OSA group was younger than the control group, with a significantly higher frequency of all features suggestive of OSA (p<0.001) and with a significant greater BMI value (p<0.001) in comparison with control subjects. The ESS score was significantly higher (p<0.001) in OSA patients than controls. No significant difference was found between OSA patients and controls regarding the sleep stages. In fact, the percentage of N1, N2, N3 and REM was similar between the groups with N1 and N2 stages being the most prevalent phases of sleep in all participants. The most important differences between OSA patients and controls were: ODI (p<0.001), AHI (p<0.001), CAP rate (cyclic alternating pattern, p<0.001) and arousal index (p = 0.002) and T90% (p<0.001).

**Table 1:** Demographic, clinical and electrophysiological data in OSA patients and controls.

Variables	*OSA patients*				*Controls*	p-value
	Total Group (n = 25)	Mild (n = 9)	Moderate (n = 10)	Severe (n = 6)	(n = 18)	
Gender (M/F)	21/4	7/2	8/2	0/6	12/6	0.48^a^
Age at examination (y)	52.5±13.7	47.2±15.9	57.4±9.4	52.2±15.2	54.4±15.9	0.48^b^
*Features suggestive of OSA*						
-Habitual loud snoring n(%)	25(100)	9(100)	10(100)	6(100)	6(33.3)	<0.001^a^
-Witnessed apnea n(%)	12(48)	2(22.2)	6(60.0)	4(66.7)	0(0)	<0.001^a^
-Nocturnal gasping or choking n(%)	17(68)	5(55.6)	7(70.0)	5(83.3)	1(5.6)	<0.001^a^
- Excessive sleepiness n(%)	23(92)	8(88.9)	9(90.0)	6(100.0)	4(22.2)	<0.001^a^
ESS score	10.4±3.6	8.7±3.7	10.3±2.8	13.0±3.5	6.2±3.4	<0.001^b^
*Abnormal ESS score (≥11*, *n%)*	14(56)	4(44.4)	5(50)	5(83.3)	3(16.7)	0.02^a^
*Medical conditions associated with increased risk for OSA*						
-BMI (Kg/m^2^)	33.2±6.6	31.5±8.2	31.7±4.6	38.2±5.0	26.4±2.8	<0.001^b^
-Hypertension n(%)	5(20)	2(8.0)	3(33.3)	0(0.0)	0(0)	0.04^a^
*Sleep Architecture*						
-N1%	32.5±12.1	34.6±16.2	31.2±9.6	31.4±10.1	32.8±9.8	0.91^b^
-N2%	50.6±10.5	45.8±7.4	52.8±12.6	54.0±9.4	47.3±6.3	0.15^b^
-N3%	13.8±10.8	15.0±10.8	14.1±12.0	11.4±10.4	15.5±7.8	0.84^b^
-REM %	3.2±3.5	4.5±3.4	2.0±3.2	3.2±3.7	4.4±3.6	0.26^c^
-Arousal Index (n/h)	10.0±6.9	4.7±5.3	11.9±6.6	14.9±4.5	3.8±4.7	<0.001^c^
-CAP Rate %	50.2±5.7	50.7±4.6	49.7±4.8	50.0±9.0	37.8±6.7	<0.001^c^
-Sleep Efficiency %	83.5±10.9	88.2±10.2	80.6±12.7	81.3±7.4	87.0±9.6	0.21^c^
-ODI (n/h)	17.5±12.9	7.7±5.4	17.3±6.6	32.4±15.4	0.7±1.0	<0.001^c^
SaO_2_ average %	92.6±2.9	93.9±1.4	93.4±2.2	89.3±3.4	94.4±1.9	0.02^c^
T 90%	8.6±14.1	2.0±1.5	6.4±8.9	22.3±22.3	0.9±1.6	<0.001^c^
-AHI	20.6±14.3	7.7±3.1	20.2±4.9	40.6±11.7	0.6±1.2	<0.001^c^

In the note of the table are reported only significant *p values* of multiple comparison tests. OSA: subjects with presence of Obstructive Sleep Apnea on polysomnography; Controls: subjects with no Obstructive Sleep Apnea on polysomnography; Data are given as mean values± standard deviations. ESS: Epworth Sleepiness Scale; BMI: body mass index; N1, stage 1 sleep; N2 stage 2 sleep; N3, stage 3 sleep; REM: REM sleep stage; %(of the sleep time). CAP: Cyclic alternating pattern; ODI: oxygen desaturation event index; T90%:Total sleep time in oxygen saturation ≤90%; AHI: apnea-hypopnea index.

^a^ Fisher’s exact test, followed by pairwise proportion test;

^b^ ANOVA test followed by pairwise t-test;

^c^ Kruskal-Wallis test followed by pairwise Wilcoxon rank sum test.

All p values are corrected according to Bonferroni. *Habitual loud snoring*: Controls vs Mild, p = 0.01; Controls vs Moderate, p = 0.008. *Witnessed apnea*: Controls vs Moderate, p = 0.008; Controls vs Severe, p = 0.009. *Nocturnal gasping or choking*: Controls vs Moderate, p = 0.009; Controls vs Severe, p = 0.007. *Excessive sleepiness*: Controls vs Mild: p = 0.024; Controls vs Moderate, p = 0.014; Controls vs Severe, p = 0.025. ESS, Controls vs Moderate, p = 0.02; Controls vs Severe, p<0.001. *BMI*: Controls vs Severe, p<0.001.

*Arousal Index*: Controls vs Moderate, p = 0.007; Controls vs Severe, p<0.011; Mild vs Severe, p = 0.046. *CAP rate*: Controls vs Mild, p = 0.002; Controls vs Moderate, p = 0.002.

*ODI*: Controls vs Mild, p<0.001; Controls vs Moderate, p<0.001; Controls vs Severe, p = 0.001; Mild vs Severe, p = 0.005. *SaO*_*2*_: Controls vs Severe, p = 0.037.

*T90%*: Controls vs Mild p = 0.045; Controls vs Severe p = 0.04; Mild vs Severe, p = 0.033.

*AHI*: Controls vs Mild, p<0.001; Controls vs Moderate, p<0.001; Controls vs Severe, p<0.001; Mild vs Moderate, p = 0.002; Mild vs Severe, p = 0.002; Moderate vs Severe, p = 0.008.

As reported in Table 1, we found no significant difference among the three OSA subgroups (mild vs moderate vs severe) regarding age, features suggestive of OSA, BMI, ESS score and sleep parameters, with the exception of ODI which showed a higher value in severe compared to mild patients (p = 0.005). By contrast, the subgroups of OSA patients were significantly different from the controls in terms of BMI, ESS score and sleep parameters (Table 1).

As shown in Table 2, over the 24-hour recording, LF and HF values did not differ significantly between the total group of OSA patients and controls. The HF and LF values of the total group of OSA patients were significantly higher during the night-time than the day-time (p<0.001), while no difference between night-time and day-time for these HRV variables was observed in the controls (Table 2). Thus, it is not surprising that the total group of OSA patients showed higher cardiac autonomic index values (as these indexes are calculated as night/day ratio) than the controls. In addition, in OSA patients, the cardiac parasympathetic index was significantly higher than the cardiac sympathetic index (p = 0.006), while both indexes were higher than in controls (parasympathetic index p<0.001, sympathetic index p<0.001).

**Table 2:** Heart rate variability parameters in OSA patients and controls.

HRV Parameters	*OSA patients*				*Controls*	p-value
	Total Group (n = 25)	Mild (n = 9)	Moderate (n = 10)	Severe (n = 6)	n = 18	
***24 Hour***						
LF, ms^2^	689.8±407.6	614.8 ± 385.2	652.3 ± 309.7	864.8 ± 581.0	695.9±405.5	0.71^a^
HF, ms^2^	627.7±373.1	684.3 ± 453.1	599.8 ± 352.0	589.1 ± 328.1	634.5±361.5	0.99^a^
***Day***						
LF, ms^2^	603.7±381.7	543.8 ± 376.4	613.4 ± 332.0	677.2 ± 511.1	699.9±389.6	0.76^a^
HF, ms^2^	464.2±277.5	489.9 ± 356.3	462.8 ± 262.4	427.8 ± 199.0	677.9±386.0	0.24^b^
***Night***						
LF, ms^2^	891.6±598.2	836.3 ± 524.0	720.3 ± 384.8	1260.2 ± 885.6	673.8±434.6	0.49^a^
HF, ms^2^	971.1±603.7	1147.2 ± 696.6	849.5 ± 531.2	909.8 ± 612.4	562.5±369.9	0.06^b^
***Cardiac Autonomic Indices***						
Cardiac Sympathetic Index (LF night/LF day)	1.37 (0.48–3.58)	1.67 (0.99–3.41)	1.31 (0.48–2.50)	1.64 (0.91–3.58)	0.92 (0.53–2.08)	0.002^a^
Cardiac Parasympathetic Index (HF night/HF day)	1.96 (1.15–5.72)	2.18 (1.33–5.72)	1.83 (1.26–2.81)	2.08 (1.15–2.84)	0.77 (0.38–1.04)	<0.001^a^

OSA: subjects with presence of Obstructive Sleep Apnea on polysomnography; Controls: subjects with absence of Obstructive Sleep Apnea on polysomnography; Data are given as mean values±standard deviations. Abbreviations: LF, low-frequency; HF, high-frequency.

^a^ Kruskal-Wallis test followed by pairwise Wilcoxon rank sum test with Bonferroni correction;

^b^ ANOVA test followed by pairwise t-test with Bonferroni correction.

LF (24 Hour) Total group vs Controls: 0.97(Mann-Whitney U test); HF (24 Hour) Total group vs Controls: 0.95 (Two sample t-test). LF (Day) Total group vs Controls: 0.5(Mann-Whitney U test); HF (Day) Total group vs Controls: 0.054

(Two sample t-testCardiac Sympathetic Index: data are given as median(range). Total group vs Controls: p<0.001(Mann-Whitney U test); Controls vs Mild, p = 0.003; Controls vs Moderate, p = 0.33; Controls vs Severe, p = 0.14; Mild vs Moderate, p = 0.57; Mild vs Severe, p = 1; Moderate vs Severe, p = 1.

Cardiac Parasympathetic Index: data are given as median(range); Total group vs Controls: p<0.001(Mann-Whitney U test); Controls vs Mild, p<0.001; Controls vs Moderate, p<0.001; Controls vs Severe, p = 0.002; Mild vs Moderate, p = 0.39; Mild vs Severe, p = 1; Moderate vs Severe, p = 1.

Night vs day in Total group of OSA: LF: p<0.001(Wilcoxon signed rank test HF: p<0.001(Wilcoxon signed rank test); Night vs day in Controls: LF: p = 0.72(one sample t-test),;HF: p = 0.005 (Wilcoxon signed rank test).

Night vs day in subgroups: Mild: LF, p = 0.003 (one sample t-test); HF, p = 0.003 (one sample t-test); Moderate: LF, p = 0.34 (one sample t-test); HF, p = 0.003 (one sample t-test); Severe: LF, p = 0.07(one sample t-test); HF, p = 0.04 (one sample t-test).

Cardiac Parasympathetic Index vs Cardiac Sympathetic Index in Total group of OSA: p = 0.006 (Wilcoxon signed rank test Cardiac Parasympathetic Index vs Cardiac Sympathetic Index in Controls: p = 0.02 (one sample t-test).

In mild, moderate and severe patients with OSA, the parasympathetic component (HF) was significantly higher in the night-time than in the daytime evaluation, whereas the sympathetic (LF) component was significantly higher in the night-time than the daytime evaluation only in patients with mild OSA. The autonomic index values in patients with mild, moderate and severe OSA were not statistically different, whereas all these values were significantly higher in comparison with the controls (Table 2).

**Fig 1:**
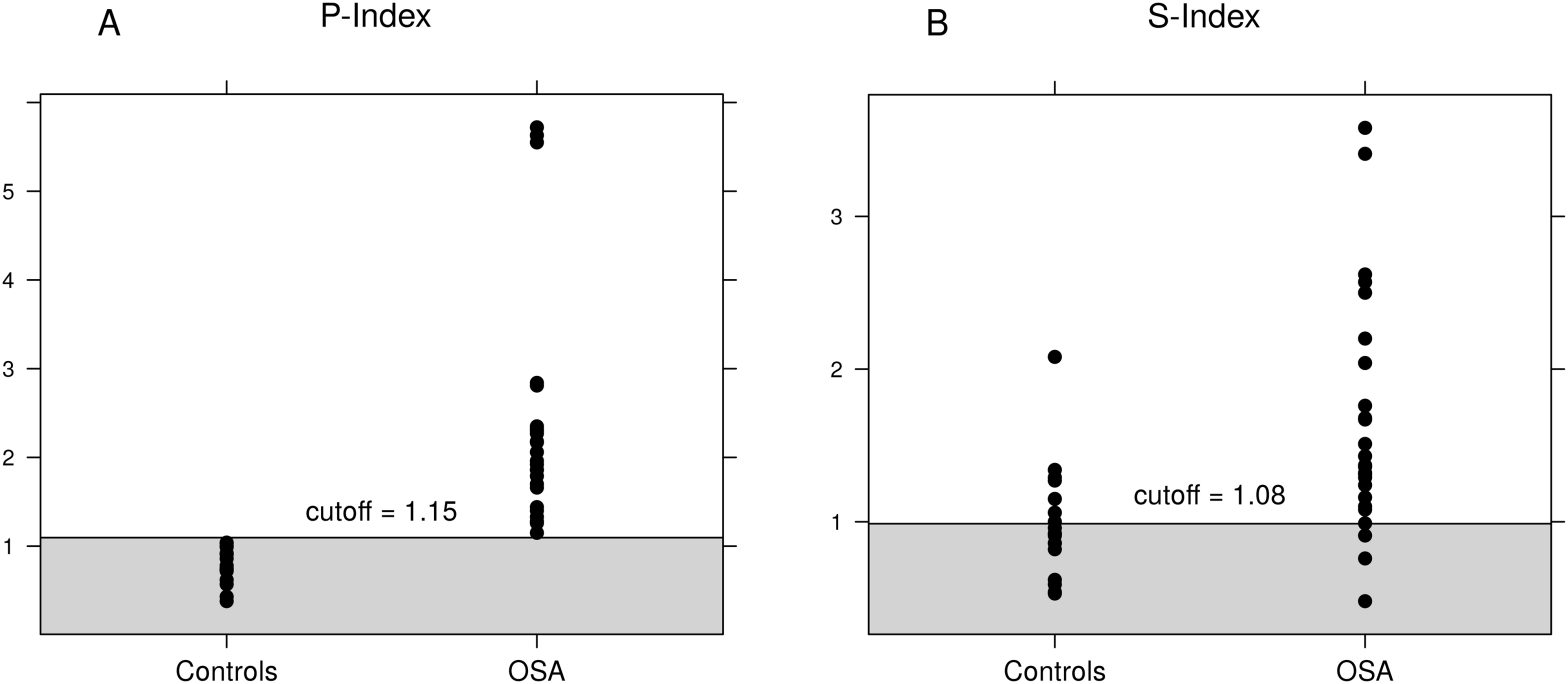
Cardiac autonomic indexes. Upper limits of cardiac parasympathetic index (P-index, 1.15, light gray area) and cardiac sympathetic index (S-index, 1.08, light gray area) in OSA patients and controls (A and B respectively). The optimal cut-off levels were obtained as the points with the highest sum of sensitivity and specificity on ROC curves.

Table 3 shows the performances of the cardiac parasympathetic and sympathetic indexes and the Epworth Sleepiness Scale for the differentiation of patients with OSA from the controls. At the cut-off level of ≥1.15, the cardiac parasympathetic index differentiated OSA patients from controls with a sensitivity, specificity and accuracy of 100%, while the cardiac sympathetic index showed a lower accuracy (79.1%) in distinguishing between the two groups. Indeed, the cardiac parasympathetic index showed no overlapping values between patients with OSA from the controls. Thus all patients with OSA were able to be correctly classified, on an individual basis (Fig 1A), whereas the cardiac sympathetic index showed an overlap of individual values between the participants from the two groups (Fig 1B).

**Table 3:** Performance of cardiac parasympathetic and sympathetic indexes and epworth sleepiness scale in differentiating OSA patients from controls.

*OSA patients vs Controls*	Cutoff	Sensitivity (%)	Specificity (%)	Accuracy (%)
**Cardiac Parasympathetic Index**	≥1.15	100	100	100
**Cardiac Sympathetic Index**	≥1.08	84	72.2	79.1
**Epworth Sleepiness Scale**	≥11	56	83.3	67.4

OSA patients: subjects with presence of Obstructive Sleep Apnea on polysomnography; Controls: subjects with absence of Obstructive Sleep Apnea on polysomnography. Optimal cutoff values were determined by using ROC curve analysis. Cardiac Parasympathetic Index vs Cardiac Sympathetic Index: p = 0.008; Cardiac Sympathetic Index vs Epworth Sleepiness Scale p = 0.36; Cardiac Parasympathetic Index vs Epworth Sleepiness Scale: p<0.001 (McNemar’s tests).

**Fig 2:**
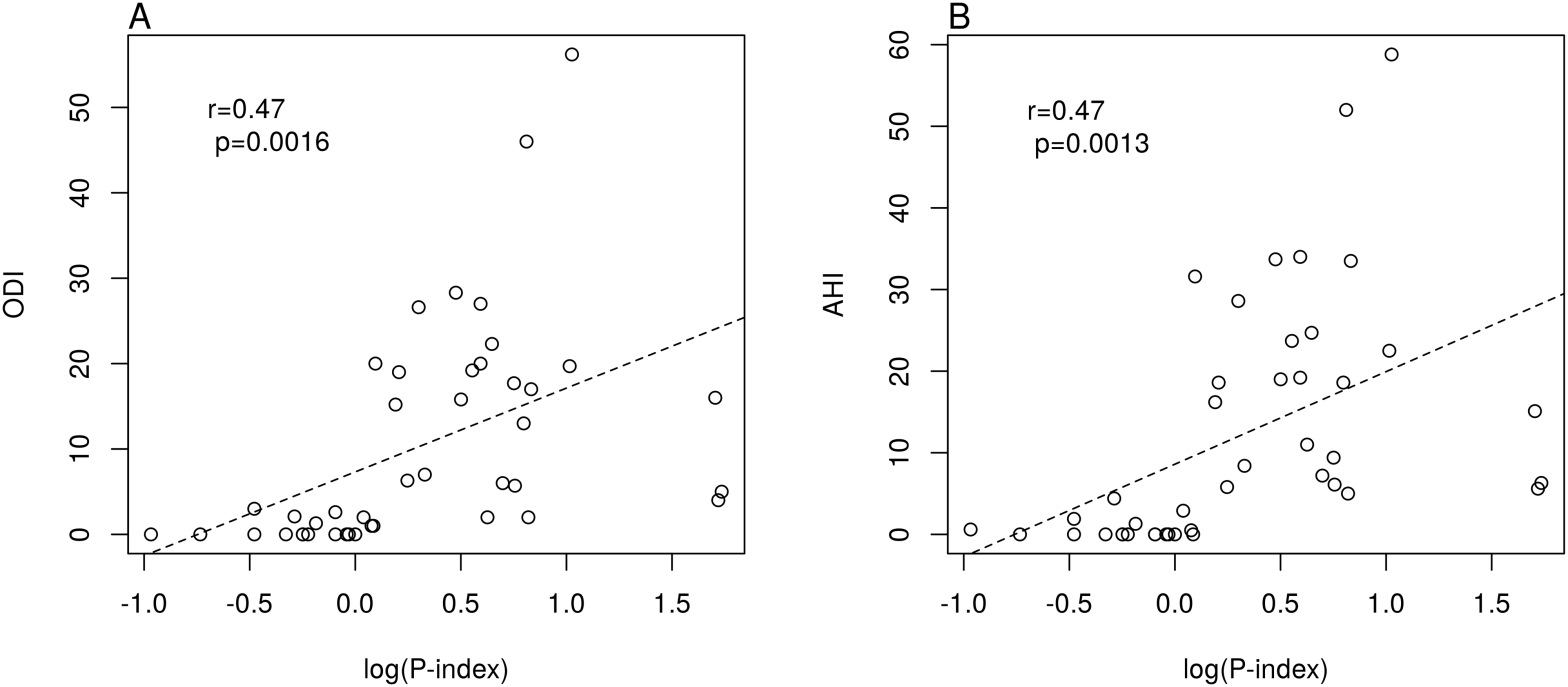
Correlation between cardiac parasympathetic index and diagnostic PSG indices. (A) Linear association between ODI and log (P-Index), Pearson correlation test: r = 0.47, p = 0.0016; linear model: intercept = 7.32, p<0.001; slope = 9.81, p = 0.0016. (B) Linear association between AHI and log(P-Index), Pearson correlation test: r = 0.47, p = 0.0013; linear model: intercept = 8.60, p<0.001; slope = 11.34, p = 0.0013.

The Epworth Sleepiness Scale had a lower accuracy (67.4%) than the cardiac autonomic indexes in differentiating OSA patients from controls (Table 3). In the whole sample, Spearman’s rank correlation showed an excellent association of the P-Index (cardiac parasympathetic index) with various PSG diagnostic indices such as ODI (rho = 0.68, p<0.0001) and AHI (rho = 0.70, p<0.0001). A good linear correlation was found between the logarithm of the P-Index and ODI (r = 0.47, p = 0.0016) (Fig 2A) and between the logarithm of the P-Index and AHI (r = 0.47, p = 0.0013) (Fig 2B).

## Discussion

To the best of our knowledge, this is the first long-term HRV study investigating cardiac autonomic fluctuations and night/day ratios of HRV power spectral components in patients with OSA in comparison with controls. Our study demonstrates that the HF spectral component of HRV was significantly higher during the night time than daytime recordings in OSA patients compared to the controls. The study also highlights that the HF night/day ratio values (cardiac parasympathetic index) accurately differentiated patients with OSA from controls on an individual basis.

In the last decade several ECG-based tools have been developed for the detection of OSA thus encouraging the use of ECG in the screening of OSA patients. Several short term spectral studies using ECG recordings with HRV analysis, a method providing information on the correct balance of ANS, have been carried out in order to evaluate not only the impact of OSA on the cardiac autonomic system, but also the usefulness of HRV analysis for OSA screening. Some authors [14] have investigated the correlation between the severity of OSA and HRV spectral components, demonstrating that HRV parameters were significantly higher in subjects with severe OSA than in those with mild OSA. Others authors [13] have focused on the correlation between HRV and PSG, showing a strong correlation between HRV parameters and AHI, MI, and ODI and suggesting that HRV examination may replace PSG monitoring among patients with OSA. Finally, other authors [24] introduced a new algorithm based on a single-lead ECG with a high accuracy in detecting subjects with OSA. In addition, heart timing has been used as a source for HRV analysis for screening patients with apnea-hypopnea syndrome [25].

Most of these studies, however, were retrospective and thus did not simultaneously analyze the HRV and PSG parameters. Some authors [15] have explored HRV parameters during sleep stages in OSA subjects, indicating that sympathovagal modulation may be impaired in these patients with parasympathetic activity tending to increase over the night.

Only two studies [16,17] have investigated HRV parameters during long-term conditions (24-hour period) in patients with OSA. Both these studies [16,17] evaluated cardiac autonomic fluctuations during a 24-hour period in the time and/or frequency domains of HRV analysis in mild and severe OSA in comparison to age-and sex-matched control subjects. These studies demonstrated that in severe OSA patients, the spectral components of HRV were significantly higher than in patients with mild OSA and controls. Taken together, this evidence suggests that autonomic activity may be altered in patients with OSA and that HRV analysis may be a powerful tool to diagnose and assess OSA. In a prospective 24-hour ECG study in young and healthy adults without known sleep-breathing disorders, significant relations were found between HRV time domain measurements and sleep apnea parameters, thus suggesting a link between autonomic dysfunction and subclinical sleep related disorders [26]. Finally, another method to screen patients with OSA using Holter ECG recording has been proposed [27]. Based on the detection of cyclic variations in heart rate (CVHR) on a tachogram, it was found that in OSA patients, longer CVHR had a positive predictive accuracy of 86% for significant sleep apnea syndrome, whereas shorter CVHR had a negative predictive accuracy of 100% [27].

Our study differs from previous studies [16,17, 26, 27] in several ways. Firstly, in our patients with OSA we performed a simultaneous HRV-PSG recording, while in the cited studies the authors retrospectively evaluated circadian apnea ECG data, HRV fluctuations or CVHR in patients with a diagnosis of OSA. Secondly, in one study [27], CVHR was quantified only for the first three hours of the Holter evaluation during the sleep stage, whereas in our study HRV parameters were evaluated over eight hours of PSG recording, during sleep and wakefulness. Thirdly, we assessed the sensitivity and specificity of the night/day ratios for LF (cardiac sympathetic index) and HF (cardiac parasympathetic index) components in differentiating OSA patients from controls. This HRV ratio has been previously demonstrated to be useful to accurately identify patients with REM sleep disorder from those without sleep disorders on an individual basis [18].

In our study, cardiac parasympathetic (HF night/day ratio) and cardiac sympathetic (LF night/day ratio) indexes were significantly higher in OSA patients than in controls, however the cardiac parasympathetic index was better at differentiating patients with OSA from controls. The cardiac parasympathetic index values in OSA patients showed no overlap with values obtained in control subjects, thus demonstrating that this index accurately differentiated patients with and without OSA on an individual basis. In fact, no patient with OSA was misdiagnosed when the cardiac parasympathetic index was used. In addition, when we stratified the OSA group of patients in mild, moderate and severe, we found that the values of the cardiac parasympathetic index were not different among the subgroups, but were significantly higher than the controls. These findings suggest that although the cardiac parasympathetic index did not differentiate among patients with mild, moderate and severe OSA, it is useful in differentiating patients with OSA from control subjects. By contrast, the cardiac sympathetic index had a sensitivity, specificity and accuracy of 84%, 72.2% and 79.1%, respectively in differentiating OSA patients from controls with some overlap of individual values between the two groups of patients. The cardiac parasympathetic index also showed a good correlation with some PSG diagnostic parameters such as ODI (r = 0.47, p = 0.0016) and AHI (r = 0.47, p = 0.0013) suggesting that this ratio was strongly related to the presence of OSA.

The ESS, a test that is indicative of subjective diurnal sleepiness, had a lower accuracy (67.4%) than the cardiac parasympathetic index in differentiating patients with OSA from controls. in fact, in our study about 44% of OSA patients had a normal ESS with a large number of false negative results. The highest percentage of false negative results on the ESS was found in patients with mild (55%) and moderate (50%) OSA. These findings are not surprising considering the low sensitivity, specificity and poor correlation of ESS with polysomnographic findings [7].

In our study, we found higher HF spectral component values during night time compared to daytime recordings in patients with OSA, which would seem to indicate an increased parasympathetic activity. The same was true in the three subgroups of OSA patients, who showed significantly higher parasympathetic activity in night-time vs daytime evaluations, whereas an increased sympathetic activity during night-time was only recorded in patients with mild OSA. By contrast, this autonomic pattern was not found in the controls, who showed similar autonomic activity during night time and daytime evaluations.

It is still debated whether in OSA patients during sleep the sympathetic or parasympathetic tone prevails. There is well documented evidence supporting the increase in sympathetic tone during sleep in OSA patients which may be explained by repetitive episodes of apnea [28,29]. The sympathetic over-activity in OSA patients, however, has also been reported during daytime wakefulness. Some authors have investigated the cardiovascular autonomic function in normotensive awake OSA patients, showing a sympathetic over-activity and a reduced baroreflex sensitivity which may predispose to arterial hypertension [30,31]. In our study, we did not find a higher sympathetic activity during daytime wakefulness probably because we used long term monitoring (16 hours) for investigating the sympathetic activity, whereas other authors [30] evaluated the sympathetic activity during short-time conditions such as the morning after a standard polygraphic sleep recording.

As for the possible involvement of parasympathetic tone during sleep in OSA patients, it has been hypothesized that in these patients the physiological shift from sympathetic to parasympathetic tone during normal sleep [32,33] might be impaired with an increase in parasympathetic modulation during NREM sleep [15]. In accordance with this hypothesis, in a large cohort of 400 OSA patients about 50% of the patients showed extreme sinus bradycardia during NREM sleep with a cyclical variation characterized by a progressive bradycardia at the onset of sleep apnea and abrupt tachycardia after the reopening of the airways and resumption of breathing [10,11]. Taken together this evidence suggests that in OSA patients there may be an increase in parasympathetic activity during NREM sleep, and that it might be an autonomic response to intermittent and repetitive episodes of apnea rather than a cardiac autonomic dysfunction in itself.

Why this occurs is not clear. One explanation may be that the apnea-induced hypoxia triggers the diving reflex through the activation of cardiac vagal tone, resulting in transient bradycardia. This protective mechanism in mammals helps to preserve blood flow to the heart and brain, limiting the demand for cardiac oxygen [8]. Bradycardia due to increased vagal activity during simulated diving has also been observed in humans [34]. Another hypothesis considers the parasympathetic over-activity during sleep as a parasympathetic overcompensation with an increase in hydraulic pressure [35]. This condition could be triggered by the carotid-aortic chemoreceptors in response to the desaturation caused by disturbed breathing, as apnea [36,37]. Some authors [38] have reported that subjects with high levels of apnea/hypopnea were associated with increased parasympathetic control during non-REM sleep stage 2, thus resulting in cardiac deceleration in this sleep stage. This compensatory mechanism is needed to stabilize the level of oxygen in the blood.

There are some limitations to our study. Firstly, the stratification of our small sample of OSA patients in mild, moderate and severe did not enable correlations to be found between HRV parameters and severity of the disease. By contrast, in the whole sample, we found a good linear correlation between the logarithm of the cardiac parasympathetic index and various PSG diagnostic indices, such as AHI and ODI. Additional studies, however, in a larger cohort are needed in order to detect the correlations between HRV parameters and severity of OSA disease.

Secondly, because we excluded patients with comorbidities or pharmacological therapy, our results cannot be generalized, and need further investigations. Moreover, HRV analysis during sleep may be modified artificially by the presence of PLMS and RBD. In our study, however, participants with these sleep disorders were excluded. Finally, there may have been environmental factors that influenced the diurnal and nocturnal recordings. In our controls, the sleep architecture was different from normative values, probably because of “first night effect”, a phenomenon commonly observed in a PSG laboratory setting [39]. Patients and controls, however, were in the same conditions as the HRV evaluations.

We believe that our study has several strengths. Firstly, HRV analysis was fully automated, thus limiting bias due to the operator-dependent technique. Secondly, the cardiac parasympathetic index accurately identified patients with OSA, and no patient was misdiagnosed when this index was used (sensitivity and specificity, 100%). Thirdly, the cardiac parasympathetic index is based on a simple and non-invasive procedure, making it useful in patients with a clinical diagnosis of OSA.

### Conclusions

Our findings indicate that the cardiac parasympathetic index may help to differentiate subjects with OSA from those without OSA, on an individual basis. Additional studies in a larger cohort are needed to further confirm the utility of the cardiac parasympathetic index in clinical practice.
